# Not a “Get Out of Jail Free Card”: Comparing the Legal Supervision of Persons Found Not Criminally Responsible on Account of Mental Disorder and Convicted Offenders

**DOI:** 10.3389/fpsyt.2021.775480

**Published:** 2022-01-18

**Authors:** Sandrine Martin, Yanick Charette, Chloé Leclerc, Michael C. Seto, Tonia L. Nicholls, Anne G. Crocker

**Affiliations:** ^1^Institut National de Psychiatrie Légale Philippe-Pinel, Montreal, QC, Canada; ^2^School of Social Work and Criminology, Université Laval, Québec, QC, Canada; ^3^School of Criminology, Université de Montréal, Montreal, QC, Canada; ^4^Forensic Research Unit, Royal Ottawa Health Care Group, Institute of Mental Health Research, Ottawa, ON, Canada; ^5^Department of Psychiatry, University of British Columbia, Vancouver, BC, Canada; ^6^British Columbia (BC) Mental Health & Substance Use Services, Coquitlam, BC, Canada; ^7^Department of Psychiatry & Addictions and School of Criminology, Université de Montréal, Montreal, QC, Canada; ^8^Research & Academics, Institut National de Psychiatrie Légale Philippe-Pinel, Montreal, QC, Canada

**Keywords:** not criminally responsible on account of mental disorder, NCRMD, justice, criminal justice system, forensic psychiatry, sentencing, insanity defense, Review Boards

## Abstract

**Background:**

The public often perceives the insanity defense as a “get out of jail free card”. Conversely, several studies demonstrate the substantial control imposed upon these defendants. This study compares Review Boards decisions regarding people found not criminally responsible on account of mental disorder (NCRMD) to criminal courts decisions regarding convicted offenders for similar offenses in Canada.

**Method:**

Detention, using logistic regression, and duration under detention and supervision, using Cox regression, were compared between a cohort of 1794 individuals found NCRMD in three Canadian provinces (Quebec, Ontario, and British Columbia) between 2000 and 2005 followed until 2008 from the National Trajectory Project and a national sample of 3,20,919 Canadians convicted of criminal offense from Statistics Canada's Criminal Court Survey.

**Results:**

Individuals found NCRMD are 3.8 times (95% CI 3.4–4.3) more likely to be detained than convicted offenders as well as 4.8 times (95% CI 4.5–5.3) and 2.9 times (95% CI 2.6–3.1) less likely to be released from detention and supervision, respectively. One year after the verdict, 73% of the NCRMD accused were still under legal supervision and 42% were still in detention, whereas these proportions were, respectively, 41 and 1% for their convicted counterparts. Interaction effects show that sex, age, jurisdiction, number of offenses, and severity of crimes committed have a differential impact on decisions applied to NCRMD accused compared to convicted persons.

**Conclusion:**

Contrary to popular perceptions, the insanity defense is not a loophole. Differences as to factors influencing the trajectories of the two samples confirm that Review Boards are able to distance their practices from the criminal courts and can set aside, at least in part, the principles of proportionality and punitiveness governing the traditional sentencing practices.

## Introduction

In many countries, a legal defense of insanity can be raised if a person commits an offense while suffering from a mental disorder that impairs their ability to appreciate that the nature and quality of their actions or omissions were wrong. In Canada, individuals who successfully raise the not criminally responsible on account of mental disorder (NCRMD) defense come under the purview of a provincial or territorial Review Board (RB) specialized in mental health. The philosophy behind RB practices is different from those that guide courts for convicted offenders. Denunciation, deterrence, and punishment are not principles that govern RB decisions as in criminal courts (1). Instead, they render dispositions according to the risk the accused represents for public safety as well as the accused's therapeutic needs (1). People found NCRMD do not receive a determinate sentence and are subject to indefinite restrictions of freedom through hospital detention or, if released, subject to conditions while living in the community until absolutely discharged.

These measures raise certain issues, debates, and questions not only from a public perception perspective (2, 3), but also from scientific and ethical perspectives. On the one hand, the NCRMD defense is associated with public perceptions that the defense is a “get out of jail free” card (4, 5), that facilitates the release of potentially dangerous individuals into the community (6–8). Conversely, several scholars stress the significant control imposed on the NCRMD population (9–12) and question the ability of RBs to distance themselves from the philosophy of the traditional penal system in their decision-making practices (13–15).

A small body of research has compared decisions and trajectories within the NCRMD population and convicted persons. NCRMD verdicts more frequently result in detention in hospital than convicted offenders (15, 16) with detention times similar to prison sentences (15–21). However, these studies were conducted more than 25 years ago; since then, significant legislative changes (22) and a recent trend toward tough crime policies have been observed (23). In addition, most of these comparisons, with a few exceptions (15, 19), did not consider the indefinite nature of NCRMD dispositions in Canada or in some states in the US in their analyses, leading to an underestimation of the length of dispositions (24, 25). Finally, these studies have compared the lengths of hospital and prison detentions, and did not take into account other restrictive community measures such as probation, for example.

The purpose of this study was therefore to assess the supervision practices and restriction of liberty imposed on people found NCRMD in comparison to people convicted of equivalent criminal offenses in Canada.

## Method

### Data

This study draws from two datasets. The first comes from the National Trajectory Project in Canada (NTP) (26) and is comprised of a cohort of individuals who received an NCRMD verdict between May 1, 2000, and April 30, 2005, in the three most populous Canadian provinces. Six individuals were lost to follow-up and were removed from the sample because no information was available for the hearing outcomes. The final sample comprises 1,794 individuals found NCRMD (Quebec, *n* = 1,089; Ontario, *n* = 483; British Columbia, *n* = 222). As Quebec had a higher number, a random sample of NCRMD verdicts, between May 1, 2000 and April 30, 2005, stratified by judicial districts, was selected. The Ontario sample was comprised of all adults with an NCRMD verdict between January 1, 2002, and April 30, 2005. The British Columbia sample was comprised of all NCRMD accused registered between May 1, 2001, and April 30, 2005. Thus, data were weighted to ensure regional representativeness (*n* = 2,661). Administrative data pertaining to outcomes (custody, supervision) were available until December 31, 2008 [*M* = 5.72 years, *SD* = 1.48, see Crocker et al. (26)]. The full NTP design and procedures are described in detail in Crocker et al. (26).

The second dataset was extracted from Statistics Canada's Criminal Courts Survey and pertained to individuals convicted between April 1, 2005 AND March 31, 2009 (27). As the observation period years were not identical for both samples, the proportion of detention decisions for the convicted sample was compared between 2000–2005 and 2005–2008 (Supplementary Table 1) to ensure that the judicial process during these two periods was similar. While these proportions between the two periods were statistically different, due to the large sample size (*N* = 300,000 to 1,400,000), the effect size observed was very small (phi <0.05). Since individuals found NCRMD could be found guilty of a new offense over the study time-period, they could theoretically end up in the convicted sample as well. It was not possible to verify if this situation occurred as Statistics Canada data are anonymized. This should not impact estimates considering the small number of NCRMD accused in comparison to the total convicted population. In order to ensure comparability with the NTP sample, only adult offenders for whom gender and age were available, from Ontario, Quebec, or British Columbia, not convicted for a type of offense not present in the NCRMD sample (e.g., prostitution, organized crime), were compared, and only the first guilty verdict during the study timeframe was retained to avoid including the same individual twice. The final sample of convicted individuals was thus comprised of 320,919 unique individuals. A description of the sample characteristics is presented in Table 1.

**Table 1:** Descriptive characteristics of the sample.

	**NCRMD** **(***N*** = 2,661)**	**Convicted** **(***N*** = 320,919)**	**Total**	
	**n/M (%/SD)**	**n/M (%/SD)**	**n/M (%/SD)**	**Khi^2^/t, d*****l, P*** **Value, phi/V/Eta**
**Offender characteristics**				
*Sex*				0.25; 1; 0.617; <0.01
Male	2248 (84.47)	269 981 (84.12)	272 229 (84.13)	
Female	413 (15.53)	50 938 (15.87)	51 351 (15.87)	
*Age (years)*				168.12; 5; <0.001; 0.02
18–29	906 (34.05)	144 478 (45.02)	145 384 (44.93)	
30–39	696 (26.15)	74 261 (23.14)	74 957 (23.16)	
40–49	635 (23.87)	65 350 (20.36)	65 985 (20.39)	
50–59	292 (10.97)	26 583 (8.28)	26 875 (8.31)	
60–69	88 (3.29)	8 206 (2.56)	8 294 (2.56)	
≥ 70	44 (1.66)	2 041 (0.64)	2 085 (0.64)	
*Province*				1903.03; 2; <0.001; 0.08
Quebec	1956 (73.51)	107 084 (33.37)	109 040 (33.70)	
Ontario	483 (18.15)	146 927 (45.78)	147 410 (45.55)	
British Columbia	222 (8.34)	66 908 (20.85)	67 130 (20.75)	
**Offense characteristics**				
Number of offenses	2.76 (2.03)	1.7 (1.47)	1.7 (1.48)	36.91; <0.001; 0.06
Severity score (log)	5.13 (1.23)	4.03 (1.11)	4.04 (1.12)	50.86; <0.001; 0.09
**Type of sentence/supervision**				
Detention^a^	1796 (67.49)	88 738 (27.65)	90 534 (27.98)	141.38; 1; <0.001; 0.02
Conditional release^a^	2155 (80.98)	193 451 (60.28)	195 606 (60.45)	473.19; 1; <0.001; 0.04
**Duration of supervision in days**				
Detention	409.49 (586.67)	29.34 (143.86)	32.46 (156.63)	127.79; <0.001; 0.22
Legal supervision	885.72 (709.35)	324.64 (365.79)	329.25 (372.40)	77.92; <0.001; 0.14

a *The sum of the percentages for the type of offense is not 100 since the categories are not mutually exclusive, a case may include more than one type of offense, as can the sum of the total detention and parole, since an individual can experience both types of measures*.

### Measures

In order to evaluate supervision and detention practices, three primary measures were used: presence of detention, time to release from detention, and time to unconditional release.

#### Presence of Detention

Detention occurs if a convicted defendant receives a sentence of imprisonment or a person is found NCRMD, a disposition of hospital custody.

#### Time to Release From Detention

Among all those who were detained at some point during the study period, time to release from detention includes, for people found NCRMD (*n* = 1,796), the sum of days between two RB hearings during which a detention decision (with or without leave permissions) was imposed. NCRMD accused still detained at the end of the study period were statistically considered as censored cases (*n* = 302), that is, the date of release was unknown. Given that only the duration issued by the court is accessible and the actual duration of detention was unknown for convicted offenders who received a detention sentence (*n* = 88,738), we used a conservative approach: we estimated detention time as 2/3 of the sentence imposed, because the vast majority are granted remission time in provincial prison (28) or statutory release in federal prison (29) if they have not already been released on parole. Since prison sentences are rarely consecutive, the longest sentence of incarceration ordered was used for analyses. For life imprisonment sentences (*n* = 123), the data were considered censored after 25 years as parole could have been granted after this period.

#### Time to Unconditional Release

Time to unconditional release considers the entire time spent under one form or another of legal supervision. For NCRMD accused, this considers the number of days between the verdict and absolute discharge (release from legal supervision of RBs without conditions). Those who did not obtain absolute discharge at the end of the observation period were censored (*n* = 512). For convicted offenders, time to unconditional release represents the number of days in prison, and/or on probation, and/or serving a sentence in the community with conditions. Convicted offenders who received a disposition other than the three aforementioned (e.g., fine; *n* = 88,388) were not included in the analyses of the length of supervision.

#### Covariates

Three available socio-demographic characteristics were included in the comparison: sex, age group (18–29, 30–39, 40–49, 50–59, 60–69, and 70+ years), and province. To assess and control for the effect of the nature of offenses, the number of offenses per sentence and the total score for the severity of crimes committed, obtained by adding the score for each offense in the verdict using Statistics Canada's offense severity index, were included (30). As this score was highly skewed to the right, a natural logarithmic transformation of the score was carried out for the analyses.

### Analyses

A logistic regression analysis was performed to compare and contrast the likelihood of detention between groups, controlling for other covariates. Given the sample included truncated observation periods for both detention and supervision times, Cox regressions were used to account for these censored data (24). Interaction effects were included in the model in order to detect the potential distinct influence of various factors on the two groups of accused individuals. Analyses were carried out using Stata 15 (31).

## Results

### Presence or Absence of Detention

People found NCRMD were 3.8 times (95% CI, 3.4–4.3) more likely to be detained than convicted offenders, even after controlling for gender, age group, province, number of offenses, and seriousness of offenses (see Supplementary Table 2 for the complete regression details).

**Figure 1:**
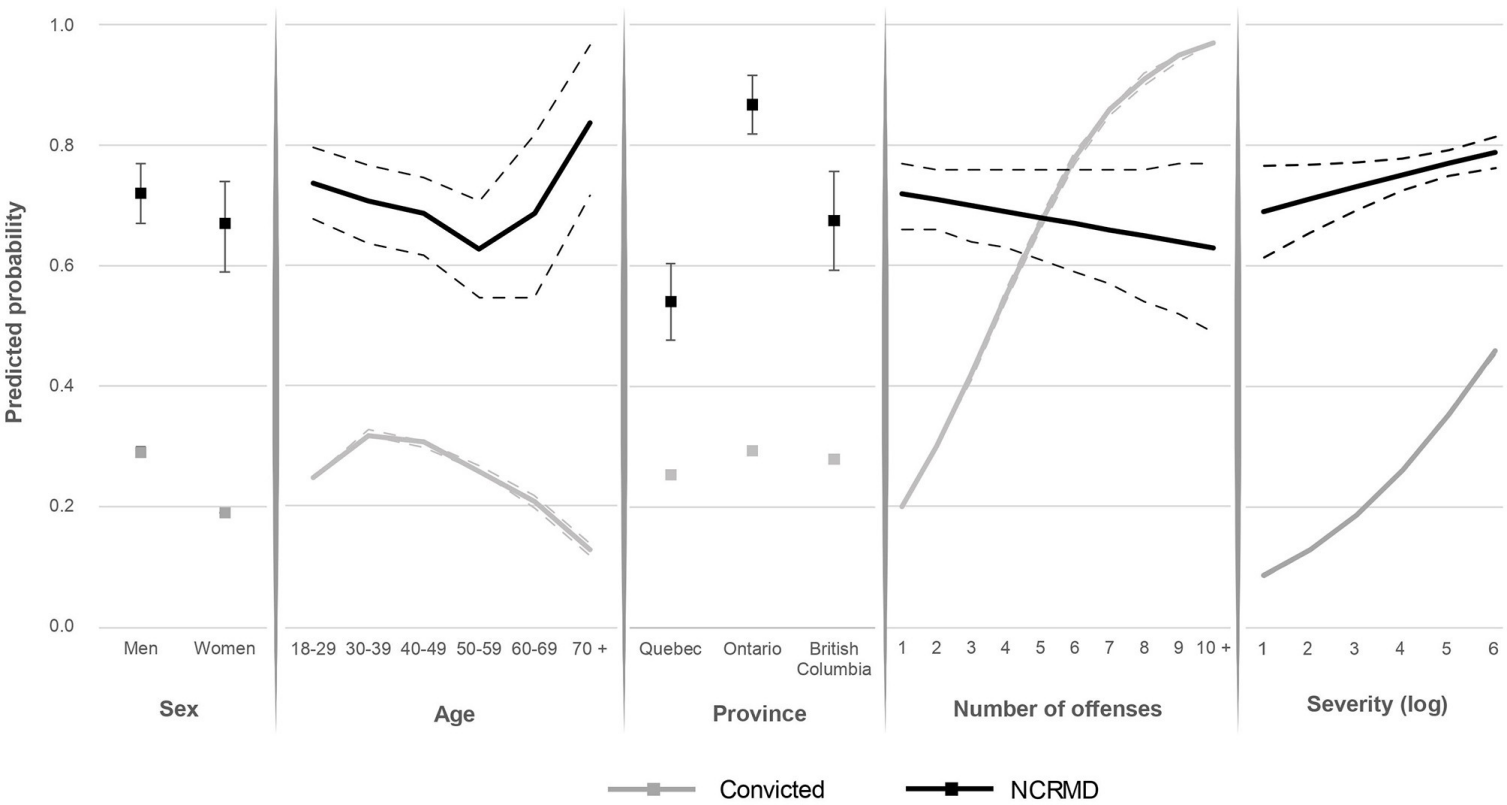
Predicted probabilities of detention for NCRMD and convicted offenders by sex, age, province, number of offenses, and offense severity^a^. – Represent confidence intervals.

Figure 1 presents the predicted probability of detention according to sex, age, province, number of offenses, and total severity score of the offense(s), for both groups. The probability of being detained was higher both for men found NCRMD and men convicted, but the sex differences were more pronounced among convicted offenders. The youngest and oldest NCRMD accused were more likely to be detained, while being between 30 and 49 years old increased the probability of detention among convicted offenders. Detention among NCRMD accused was more common in Ontario than in Quebec. The number of offenses included in the verdict increased the probability of detention among convicted offenders only. Finally, having been charged with more severe offenses indicated a higher probability of detention for convicted offenders and, although to a lesser extent, for NCRMD accused. For example, for a crime such as disturbing the peace, the estimated likelihood of being detained in custody was 55% for convicted individuals in the sample while it was 81% for NCRMD individuals. In the case of a homicide, the estimated likelihood of being detained was 88% for both groups.

### Time to Release From Detention

**Figure 2:**
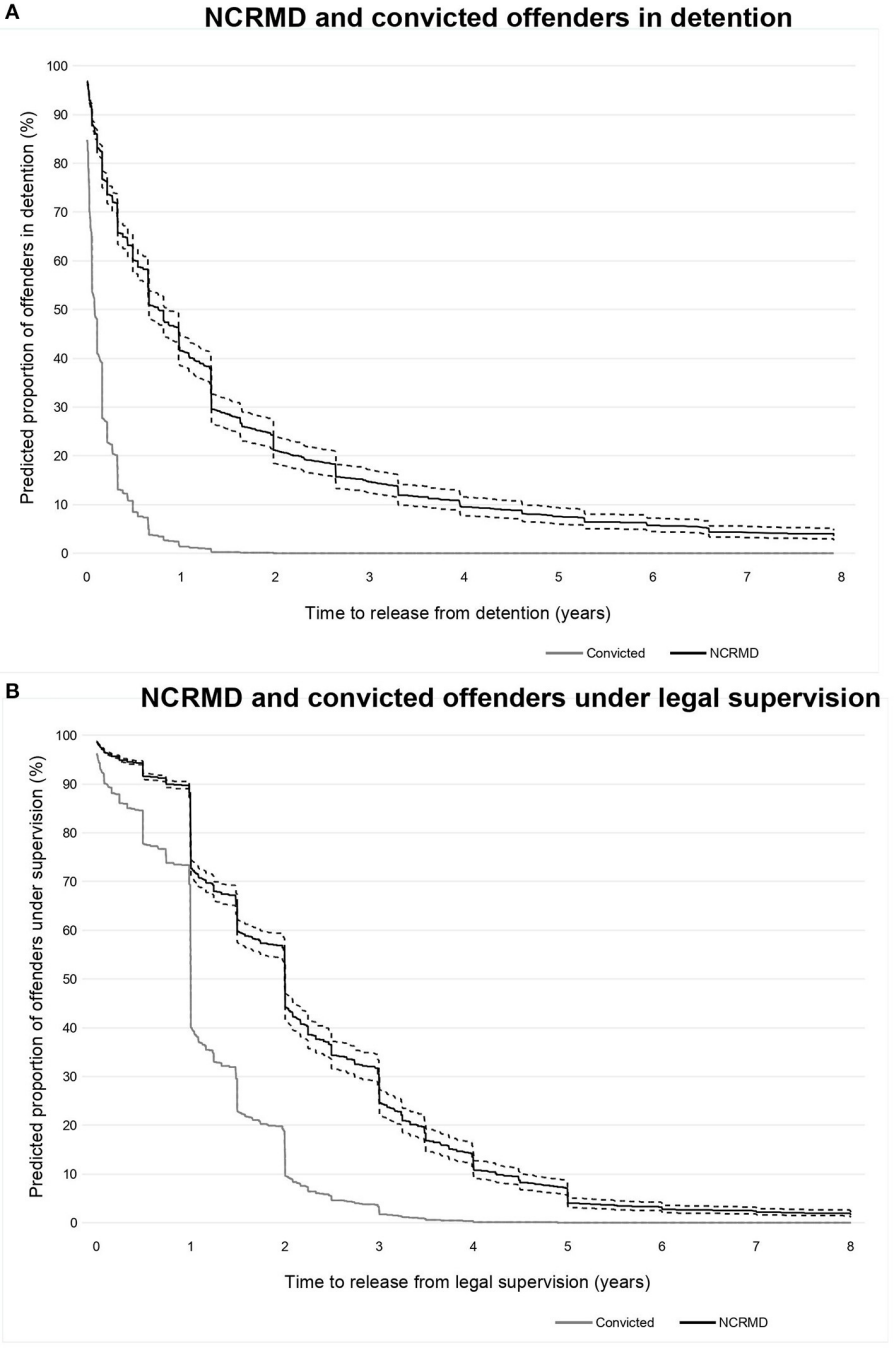
**(A,B)** Predicted proportion of NCRMD and convicted offenders in detention and under legal supervision over the years following a verdict^*a*^. – Represent confidence intervals.

NCRMD accused were 4.8 times (95% CI, 4.5–5.3) less likely to be discharged from detention than convicted offenders with similar characteristics (see Supplementary Table 2). Figure 2A presents the likelihood of still being detained over time controlling for other covariates. One year following a verdict, 42% of the NCRMD accused were still detained, while 1% of convicted offenders in the sample were still in custody. After three years, these proportions were 15% and almost nil (0.01%), respectively.

**Figure 3:**
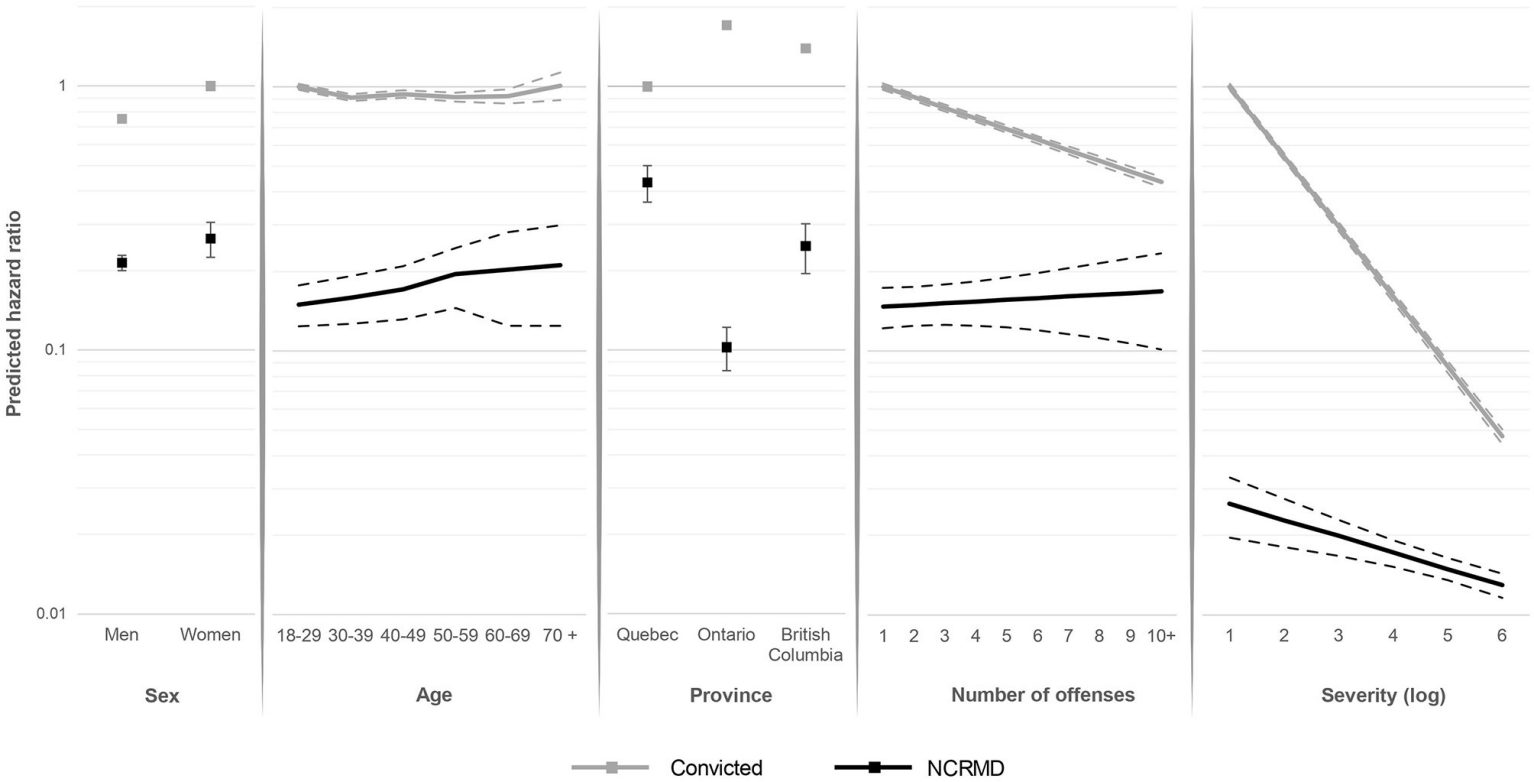
Predicted odds of being released from detention over time for NCRMD and convicted offenders by sex, age, province, number of offenses, and offense severity^*a*^. – Represent confidence intervals.

Figure 3 presents the hazard ratio of being released from detention over time according to sex, age, province, number of offenses, and total severity score of the offense(s), for both groups. Being male increased time to release for both groups. Younger NCRMD accused were less likely to be released, compared to older NCRMD accused, while younger convicted offenders (18–29 years) were the most likely to be released, even if age had a smaller effect on convicted offenders. Convicted offenders in Quebec had longer detention durations than those from Ontario and British Columbia, while the opposite was observed for individuals found NCRMD (shorter detention duration in Quebec compared to the other two provinces). A greater number of offenses included in the verdict, as well as more severe offenses, significantly decreased the probability of release from detention among convicted offenders, while these factors had minor effects among NCRMD accused.

### Time to Unconditional Release/Absolute Discharge

For each day spent under legal supervision, NCRMD accused were 2.9 times (95% CI, 2.6–3.1) less likely to attain absolute discharge compared to convicted offenders after having controlled for sex, age, province, number, and seriousness of offense(s) (see Supplementary Table 2). Figure 2B presents the likelihood of still being under legal supervision over time after a verdict, for NCRMD accused and convicted offenders, controlling for other covariates. After one year following a verdict, 73% of the NCRMD accused were still under the purview of the RB, while 41% of convicted offenders in the sample still had legal supervision after that time. After 3 years, these proportions were 45 and 2%, respectively.

**Figure 4:**
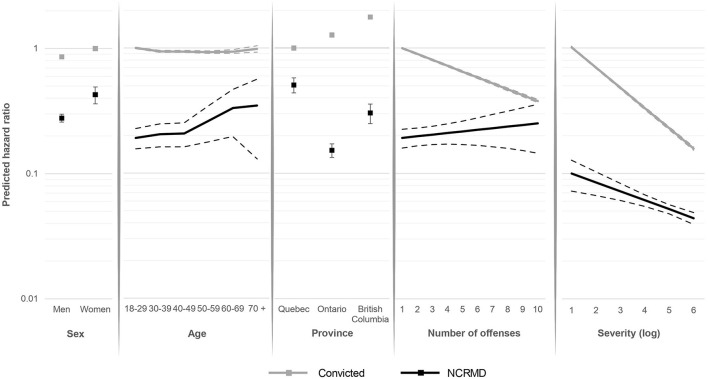
Predicted odds of being released from supervision over time for NCRMD and convicted offenders by sex, age, province, number of offenses, and offense severity^a^. – Represent confidence intervals.

Figure 4 presents the hazard ratio of being released from legal supervision over time according to sex, age, province, number of offenses, and total severity score of the offense(s), for both groups. Men were less likely than women to obtain an absolute discharge in both groups although this effect is larger among NCRMD accused. Younger NCRMD accused (18–29 years) were also less likely to be released than other age groups, whereas the youngest group was the most likely to be released from judicial supervision among convicted offenders, even if this effect is minor. Having a verdict in Quebec reduced the likelihood of being released for convicted offenders but increased this probability for people found NCRMD. Finally, while a higher number of offenses, as well as having committed more severe offenses, significantly decreased the probability of obtaining an absolute discharge among convicted offenders, these effects were weaker and almost absent for the number of offenses in the NCRMD sample.

## Discussion

Results showed that, on average, NCRMD accused received detention dispositions more frequently and had longer periods of detention and supervision than convicted offenders, even after controlling for the effects of gender, age, province, number of offenses, and seriousness of offenses.

The fact that NCRMD accused are subject to a higher level of legislated control and have their civil liberties restricted for significantly longer when compared to convicted offenders highlights the clear gap between political, public, and media discourse and actual practice. These results are in line with several American and Canadian studies showing non-evidence-based perceptions (32, 33). Contrary to perceptions about the NCRMD defense being a “get out of jail free card,” the reality is that NCRMD accused undergo considerable periods of detention and supervision, as compared to their convicted counterparts. Of particular relevance, the forensic population has low rates of reoffending (34–38) and revocation of release (35–40) [possibly because of the access to psychiatric care and the supervision provided by RB decisions, including a psychiatric follow-up in the community provided during conditional release (38, 41) and conditions allowing hospitalization in case of re-emergence of symptoms (10, 42)], and thus represents a lower risk to the public than people who are convicted. This divergence is relevant to inform the debate around the NCRMD defense, more generally (43) and is especially important considering the more punitive trends observed in the beginning of the 2000s in sentencing legislation (23)and more specifically with the Not Criminally Responsible Reform Act (Law C-14) in 2014 which created the high-risk accused designation and stated that public safety is the predominant decision criterion, before the therapeutic needs of the individual (13, 22). Our findings highlight the importance of translating and disseminating accurate information regarding this defense, especially since some studies in the United States have indicated that negative attitudes toward the defense attenuated when presented with facts (33, 44).

These results also show the consequences that indeterminate dispositions can have on individuals, which can include a long period of supervision, making the defense less advantageous than it appears at first glance if time under supervision or in detention is the primary consideration. This observation is in line with qualitative research with people found NCRMD themselves who emphasize the long periods of supervision (45, 46). These prolonged periods of supervision also have societal implications, imposing financial pressures due to the cost of inpatient forensic beds (40) and requiring considerable resources of the health system which limit those allocated to general psychiatry, a phenomenon noted on an international scale (47). The money spent on the detention and supervision of individuals found NCRMD could probably be spent more efficiently in prevention programs and/or supported housing (39, 48), addressing the issue at the source.

As previously observed (10, 49, 50), disparities across jurisdictions exist with respect to the measures imposed on people found NCRMD, raising some questions about the equivalent treatment of NCRMD accused across Canadian jurisdictions. Some explanations regarding these disparities might include the larger pool of NCRMD individuals in Quebec (9), which might encourage a faster release decision to free up resources, and the frequent use of a hybrid disposition of “detention with conditions” in Ontario, equivalent to conditional discharge in other regions with arrangements for release to a specific community location (10), Differences in the supervision trajectories of the two groups also exist as to the influence of age and sex. The differences in terms of the influence of age could be explained by the fact that convicted offenders in the age groups between 30 and 50 years old are more likely to have a criminal history and therefore undergo longer supervision and more detention disposition, while younger NCRMD defendants may require more supervision since they are possibly less known by psychiatric services and have not yet received the appropriate treatment (optimal treatment not identified, patient with less insight, drug abuse not addressed). Being in an older age group can be considered a mitigating factor in sentencing for convicted offenders (51) while older NCRMD defendants may require more supervision due to age-related neuro-cognitive disorders (e.g., dementia) which can complicate the treatment. Sex influences the trajectories of the two groups in the same way but has a greater effect on the length of supervision for NCRMD individuals, and on detention disposition for convicted offenders. These differences in effect size could be related to the attention paid to risk evaluation over time for NCRMD individuals (being male increasing the risk of recidivism). That being said, the differences in the supervision trajectories exist especially with respect to the seriousness of the offense(s). RB decisions are influenced by the nature of the offense, but to a far less extent than for the criminal courts. Thus, RBs can set aside, at least in part, the principles of proportionality and punitiveness governing the traditional criminal justice system sentencing practices.

### Limitations

The differences presented between the supervision of NCRMD and convicted offenders relate only to the durations and the type of dispositions, while it is possible that the two groups experience their supervision very differently. The NCRMD accused are in a therapeutic environment within the health care system, while the convicted defendants are in a more punitive correctional system. Prison could prove to be a particularly harmful environment and the criminal record burdensome, while the therapeutic management of people found NCRMD can involve significant and intrusive control, because the person found NCRMD must comply with treatment and other requirements in order to be released from detention and eventually from any legal supervision. The indeterminate aspect of supervision for NCRMD accused can also be very difficult for them (45, 46, 52). On the other hand, NCRMD individuals who receive detention with leave permission can access the community if the medical team agrees, and convicted offenders can be granted parole. It would therefore be interesting to complement our results through qualitative interviews in each group to see how the outcomes of these two different verdicts are experienced.

Additional limitations related to the data such as the fact that the years covered for the convicted sample (2005–2008) did not match up perfectly with the NCRMD sample (2000–2008) should be noted, although no major changes in the legislations or penal practices were observed between these periods. Furthermore, this study was carried out in only three Canadian provinces, the results therefore relate only to a part, albeit the majority, of people found NCRMD in Canada. In addition, we were limited to the information available in both datasets. For example, we know that ethnicity might influence sentencing processes (53), but this information was not available in the convicted offenders' dataset. Similarly, criminal history data were not available for convicted offenders, although this would be expected to have an impact on sentencing. Controlling for prior convictions could possibly enhance the difference between the duration of detention of the two groups since a large proportion of NCRMD accused had no prior offenses (54). It should also be mentioned that data on suspended sentences were not available in Quebec, which can reduce the time measured for convicted defendants in this province, though the impact was likely negligible, given the fact that this measure represents only 4% of sentences in Canada (55). Finally, since legislative changes in the penal system (such as Law C-10, 2012) and in the law governing NCRMD accused (with Law C-14, 2014) have occurred, it would be appropriate, in future studies, to integrate data beyond 2008 to assess the consequences of these changes on the practices of the systems in which these accused operate. Even though the data are from 10 years ago, these results are among the most recent available large-scale datasets on the topic.

## Conclusion

In the light of this large sample study allowing a contemporary comparison between the trajectories of individuals found NCRMD and convicted offenders, it is possible to see, as in previous studies, that the insanity defense is not a loophole, but also that more restrictive laws being adopted regarding this vulnerable population are unnecessary. Finally, significantly more effort needs to be put into developing prevention strategies for persons whose offenses are intrinsically linked to their symptomatology and who end up in institutions and under legal supervision, for a significant amount of time.

## Data Availability Statement

Access to data from the Criminal Courts Survey is not available since access to this data is protected by Statistics Canada. To ensure anonymity of participant, access to NTP data is not possible as it includes sensitive information. Requests to access the datasets should be directed to None, sandrine.martin.2@umontreal.ca.

## Author Contributions

SM, YC, and CL devised the study concept. SM did the data analysis with input from YC and CL. SM and YC produced the figures. SM wrote the first draft of the manuscript, had full access to all the data in the study and takes responsibility for the integrity of the data and the accuracy of the data analysis. All authors contributed to editing and commenting on the final version.

## Funding

This research was made possible through a grant (#435-2017-0759) to CL from Social Sciences and Humanities Research Council (SSHRC) and a grant (#RSQ 9962 Volet B) from Fonds de Recherche Quebec (FRQ) and the Mental Health Commission of Canada to AC. SM acknowledges the support of the SSHRC for a Master's fellowship (#766-2018-0242). YC acknowledges the support of FRQ in the form of a salary award (#2019.253929).

## Conflict of Interest

The authors declare that the research was conducted in the absence of any commercial or financial relationships that could be construed as a potential conflict of interest.

## Publisher's Note

All claims expressed in this article are solely those of the authors and do not necessarily represent those of their affiliated organizations, or those of the publisher, the editors and the reviewers. Any product that may be evaluated in this article, or claim that may be made by its manufacturer, is not guaranteed or endorsed by the publisher.
