# Supplementary material for: Not a “Get Out of Jail Free Card”: Comparing the Legal Supervision of Persons Found Not Criminally Responsible on Account of Mental Disorder and Convicted Offenders
Source: Front Psychiatry. 2022 Jan 18;12:775480. doi: 10.3389/fpsyt.2021.775480 (PMC8804320; doi:10.3389/fpsyt.2021.775480)
Supplement: Supplementary file 1 [file Data_Sheet_1.docx]

Supplementary Material

# Table 1: Proportions of detention by offense category in 2000-2005 and 2005-2009

| **Offense** | **Detention** | **Years** | | **Khi^2^,**  **P Value, phi** |
| --- | --- | --- | --- | --- |
|  |  | *2000-2005*  *n(%)* | *2005-2008*  *n(%)* |  |
| Crimes against the person | Yes | 112 692 (15.69)  605 662 (84.31) | 89 362 (16.48) | 145.77; <.001; 0.01 |
|  | No |  | 452 736 (83.52) |  |
| Crimes against property | Yes | 170 274 (21.22) | 139 246 (23.27) | 835.33; <.001; 0.02 |
|  | No | 632 164 (78.78) | 459 208 (76.73) |  |
| Crimes against the administration of justice | Yes | 144 330 (31.90) | 135 966 (33.63) | 288.12; <.001; 0.02 |
|  | No | 308 064 (68.10) | 268 368 (66.37) |  |
| Other criminal Code offenses | Yes | 27 808 (18.17) | 24 312 (21.52) | 462.15; <.001; 0.04 |
|  | No | 125 228 (81.83) | 88 674 (78.48) |  |

# Table 2 – Regression models predicting the presence or absence of detention, time to release from detention and time to complete release

|  | **Model #1**  **Logistic Regression** | | | **Model #2**  **Cox Regression** | | | **Model #3**  **Cox Regression** | | |
| --- | --- | --- | --- | --- | --- | --- | --- | --- | --- |
|  | Presence or absence of detention | | | Release from detention | | | Release from supervision | | |
|  | (n = 323 580) | | | (n = 90 534) | | | (n = 235 192) | | |
|  | *Exp(b)* | *95% CI* | *P Value* | *RR* | *95% CI* | *P Value* | *RR* | *95% CI* | *P Value* |
| **Type of verdict**  **(ref : convicted)** |  |  | |  |  |  |  |  |  |
| NCRMD | 3.82 | (3.42-4.26) | <.001 | 0.21 | (0.19-0.22) | <.001 | 0.35 | (0.32-0.38) | <.001 |
| **Sex** |  |  |  |  |  |  |  |  |  |
| Men | 1.94 | (1.89-1.99) | <.001 | 0.79 | (0.77-0.81) | <.001 | 0.91 | (0.91-0.92) | <.001 |
| **Age (ref: 18-29 years)** |  |  |  |  |  |  |  |  |  |
| 30-39 | 1.53 | (1.50-1.57) | <.001 | 0.84 | (0.83-0.86) | <.001 | 0.89 | (0.88-0.90) | <.001 |
| 40-49 | 1.44 | (1.41-1.47) | <.001 | 0.79 | (0.77-0.80) | <.001 | 0.85 | (0.85-0.86) | <.001 |
| 50-59 | 1.14 | (1.10-1.18) | <.001 | 0.69 | (0.67-0.71) | <.001 | 0.82 | (0.81-0.84) | <.001 |
| 60-69 | 0.80 | (0.76-0.86) | <.001 | 0.67 | (0.63-0.71) | <.001 | 0.82 | (0.79-0.84) | <.001 |
| > 70 | 0.44 | (0.38-0.51) | <.001 | 0.81 | (0.72-0.91) | <.001 | 0.84 | (0.79-0.89) | <.001 |
| **Province (ref: Quebec)** |  |  |  |  |  |  |  |  |  |
| Ontario | 1.47 | (1.42-1.50) | <.001 | 1.64 | (1.61-1.67) | <.001 | 1.18 | (1.17-1.19) | <.001 |
| British Columbia | 1.43 | (1.39-1.46) | <.001 | 1.47 | (1.44-1.49) | <.001 | 1.73 | (1.71-1.76) | <.001 |
| Number of offenses | 1.35 | (1.34-1.36) | <.001 | 1.10 | (1.10-1.11) | <.001 | 1.01 | (1.01-1.01) | <.001 |
| Severity score (log) | 1.59 | (1.39-1.61) | <.001 | 0.55 | (0.54-0.56) | <.001 | 0.69 | (0.69-0.70) | <.001 |
| Constant | 0.01 | (0.01-0.01) | <.001 |  |  |  |  |  |  |
| R^2^ Nagelkerke | 12.80% | | | 35.25% | | | 18.51% | | |
| Log likelihood | -166 543.74 | | | -922 055.58 | | | -2 654 328.10 | | |
| Khi^2^ (dl), p | 48 902.57 (11) <.001 | | | 20 432.18 (11) <.001 | | | 43 173.96 (11) <.001 | | |
|  | | | | | | | | | |

**Table 2 (continued) :**

|  | **Model #4**  **Logistic regression with the effect of interaction of the group of accused** | | | **Model #5**  **Cox Regression with the effect of interaction of the group of accused** | | | **Model #6**  **Cox Regression with the effect of interaction of the group of accused** | | |
| --- | --- | --- | --- | --- | --- | --- | --- | --- | --- |
|  | Presence or absence of detention | | | Probability of release from detention | | | Probability of release from supervision | | |
|  | (n = 323 580) | | | (n = 90 534) | | | (n = 235 192) | | |
|  | *Exp(b)* | *95% CI* | *P Value* | *RR* | *95% CI* | *P Value* | *RR* | *95% CI* | *P Value* |
| **Type of verdict (ref : Convicted)** |  |  | |  |  |  |  |  |  |
| NCRMD | 72.88 | (41.45-  128.15) | <.001 | 0.05 | (0.03-0.07) | <.001 | 0.22 | (0.15-0.33) | <.001 |
| **Sex** |  |  |  |  |  |  |  |  |  |
| Men | 1.95 | (1.90-2.00) | <.001 | 0.79 | (0.77-0.81) | <.001 | 0.92 | (0.91-0.93) | <.001 |
| **Age (ref: 18-29 years)** |  |  |  |  |  |  |  |  |  |
| 30-39 | 1.54 | (1.51-1.57) | <.001 | 0.84 | (0.83-0.85) | <.001 | 0.89 | (0.88-0.90) | <.001 |
| 40-49 | 1.45 | (1.42-1.48) | <.001 | 0.78 | (0.77-0.80) | <.001 | 0.85 | (0.84-0.86) | <.001 |
| 50-59 | 1.15 | (1.11-1.19) | <.001 | 0.68 | (0.66-0.70) | <.001 | 0.82 | (0.80-0.83) | <.001 |
| 60-69 | 0.80 | (0.76-0.86) | <.001 | 0.66 | (0.62-0.70) | <.001 | 0.81 | (0.79-0.83) | <.001 |
| > 70 | 0.41 | (0.35-0.48) | <.001 | 0.74 | (0.66-0.83) | <.001 | 0.82 | (0.78-0.87) | <.001 |
| **Province (ref: Quebec)** |  |  |  |  |  |  |  |  |  |
| Ontario | 1.46 | (1.44-1.49) | <.001 | 1.69 | (1.66-1.72) | <.001 | 1.19 | (1.18-1.20) | <.001 |
| British Colombia | 1.43 | (1.39-1.46) | <.001 | 1.50 | (1.47-1.53) | <.001 | 1.75 | (1.73-1.78) | <.001 |
| Number of crimes | 1.35 | (1.34-1.36) | <.001 | 1.11 | (1.10-1.11) | <.001 | 1.01 | (1.01-1.01) | <.001 |
| Severity score (log) | 1.59 | (1.58-1.61) | <.001 | 0.54 | (0.54-0.55) | <.001 | 0.69 | (0.69-0.69) | <.001 |
| NCRMD # Men | 0.66 | (0.50-0.88) | .004 | 0.84 | (0.70-1.01) | .066 | 0.61 | (0.50-0.75) | <.001 |
| NCRMD # 30-39 years | 0.53 | (0.40-0.70) | <.001 | 1.30 | (1.08-1.57) | .005 | 1.22 | (0.99-1.49) | .055 |
| NCRMD # 40-49 years | 0.47 | (0.36-0.63) | <.001 | 1.45 | (1.17-1.78) | .001 | 1.28 | (1.03-1.59) | .025 |
| NCRMD # 50-59 years | 0.48 | (0.36-0.70) | <.001 | 1.96 | (1.54-2.49) | <.001 | 1.75 | (1.27-2.41) | .001 |
| NCRMD # 60-69 years | 0.76 | (0.42-1.37) | .358 | 2.23 | (1.50-3.30) | <.001 | 2.50 | (1.69-3.70) | <.001 |
| NCRMD # 70 years + | 5.60 | (2.05-15.29) | .001 | 1.82 | (1.18-2.82) | .007 | 2.13 | (1.16-3.92) | .015 |
| NCRMD # Ontario | 4.57 | (3.29-6.37) | <.001 | 0.13 | (0.11-0.15) | <.001 | 0.24 | (0.20-0.29) | <.001 |
| NCRMD # Columbia | 1.36 | (0.98-1.89) | .067 | 0.37 | (0.31-0.45) | <.001 | 0.35 | (0.28-0.43) | <.001 |
| NCRMD #Number of offenses | 0.73 | (0.68-0.78) | <.001 | 0.94 | (0.90-0.98) | .001 | 1.01 | (0.97-1.06) | .595 |
| NCRMD # Severity score | 0.71 | (0.64-0.78) | <.001 | 1.60 | (1.51-1.69) | <.001 | 1.23 | (1.15-1.31) | <.001 |
| Constant | 0.01 | (0.01-0.01) | <.001 |  |  |  |  |  |  |
| R^2^ Nagelkerke | 12.90% | | | 26.26% | | | 18.79% | | |
| Log likelihood | -166 351.38 | | | -921 353.58 | | | -2 653 911.50 | | |
| Khi^2^ (dl), p | 49 287.29 (21) <.001 | | | 20 878.53 (21) <.001 | | | 44 774.35 (21) <.001 | | |
|  | | | | | | | | | |

**
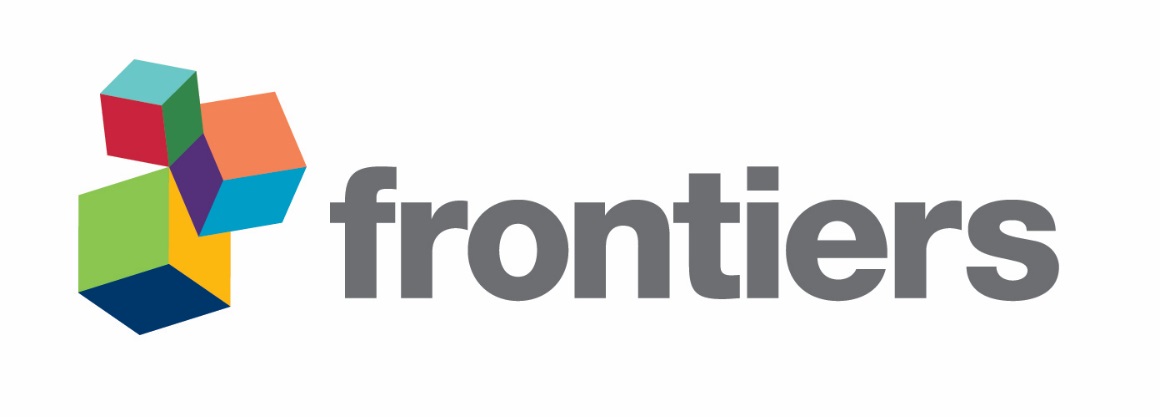
**
